# Impact of Pus1 Pseudouridine Synthase on Specific Decoding Events in *Saccharomyces cerevisiae*

**DOI:** 10.3390/biom10050729

**Published:** 2020-05-07

**Authors:** Bahar Khonsari, Roland Klassen

**Affiliations:** Institut für Biologie, Fachgebiet Mikrobiologie, Universität Kassel, Heinrich-Plett-Str. 40, D-34132 Kassel, Germany; bahar.khonsari@gmail.com

**Keywords:** tRNA modification, *PUS1*, pseudouridine, translation, non-sense suppression, *sup70-65*, misreading, rapid tRNA decay

## Abstract

Pus1-dependent pseudouridylation occurs in many tRNAs and at multiple positions, yet the functional impact of this modification is incompletely understood. We analyzed the consequences of *PUS1* deletion on the essential decoding of CAG (Gln) codons by tRNA^Gln^CUG in yeast. Synthetic lethality was observed upon combining the modification defect with destabilized variants of tRNA^Gln^CUG, pointing to a severe CAG-decoding defect of the hypomodified tRNA. In addition, we demonstrated that misreading of UAG stop codons by a tRNA^Gln^CUG variant is positively affected by Pus1. Genetic approaches further indicated that mildly elevated temperature decreases the decoding efficiency of CAG and UAG via destabilized tRNA^Gln^CAG variants. We also determined the misreading of CGC (Arg) codons by tRNA^His^GUG, where the CGC decoder tRNA^Arg^ICG contains Pus1-dependent pseudouridine, but not the mistranslating tRNA^His^. We found that the absence of Pus1 increased CGC misreading by tRNA^His^, demonstrating a positive role of the modification in the competition against non-synonymous near-cognate tRNA. Part of the in vivo decoding defects and phenotypes in *pus1* mutants and strains carrying destabilized tRNA^Gln^CAG were suppressible by additional deletion of the rapid tRNA decay (RTD)-relevant *MET22*, suggesting the involvement of RTD-mediated tRNA destabilization.

## 1. Introduction

Pseudouridine (Ψ) represents the most abundant tRNA modification and can be found within the acceptor stem, D-stem, anticodon stem loop, and the T-loop of tRNA [1,2,3]. Multiple Ψ synthases are involved in the modification of specific positions, and defects in several of them are linked to human disease [3,4,5]. In yeast, the loss of individual Ψ synthases may affect growth rate, global transcription of amino acid biosynthesis genes, and amino acid levels as well as lipid content [6,7,8,9]. Pus1 is a multisite Ψ synthase that modifies tRNA positions 1,26,27,28,34,36,65, and 67 [10,11,12,13]. Despite the fact that several tRNAs are naturally modified by Pus1, some at multiple positions [14], yeast can tolerate the loss of Pus1 without growth defects at optimum temperature [11]. This suggests that under this condition, yeast translation operates at nearly normal efficiency even in the combined absence of all Pus1-dependent modifications. However, elevated temperature causes a noticeable growth delay of *pus1*-mutant cells [15]. In addition, *pus1* mutation is known to result in synthetic lethality in yeast when combined with the loss of a second Ψ synthase (Pus4), nuclear tRNA export protein Los1, or in cells carrying a mutant allele of tRNA^Gln^CUG (*slc58*) [11,16]. These genetic interactions suggest that Pus1-dependent pseudouridylation becomes critical when other aspects of tRNA biogenesis or modification are disturbed. Strong negative genetic interactions of various tRNA modification genes in general support the idea of cooperativity and functional redundancy between different modifications, which may explain the weak phenotypes observed for many single-modification defects [15,17,18,19,20,21,22,23]. In humans, loss of Pus1 function is linked to the disorder MLASA (mitochondrial myopathy with lactic acidosis and sideroblastic anemia), which underscores the general importance of Pus1-dependent Ψ formation [24].

A temperature-sensitive (*ts*) growth defect is observed in several tRNA modification mutants [15,18,19,20]. In some cases, such a phenotype is caused by the temperature-dependent induction of a tRNA surveillance mechanism termed rapid tRNA decay (RTD) [18,25]. Activation of RTD at elevated temperatures results in highly specific destabilization of individual tRNAs, and the concomitant tRNA depletion causes the inability to grow in this condition. RTD involves exonucleases Rat1 and Xrn1 and is strongly inhibited upon deletion of *MET22*, a gene involved in sulfur assimilation [26]. The inhibitory function of *met22* mutation occurs due to the accumulation of pAp (adenosine 3′,5′ bisphosphate), a sulfur assimilation byproduct inhibiting both of the RTD-relevant exonucleases [26,27]. Hence, *met22* mutation suppresses growth defects of several tRNA modification mutants linked to RTD and inhibits the active destabilization of specific tRNAs [26].

To better characterize the in vivo role of Pus1 in tRNA function, we tested the requirement of the synthase for specific decoding events in yeast and determined the dependency of phenotypes associated with *pus1* mutation or destabilized tRNA^Gln^CUG variants on the RTD factor *MET22*. We demonstrated that two additional variants of tRNA^Gln^CUG caused synthetic lethality when combined with a *pus1* mutation. This effect, as well as individual phenotypes of the modification defect and the destabilized tRNA, were suppressed in the absence of *MET22*. Furthermore, specific decoding defects occurred in cells lacking Pus1-dependent pseudouridylation, and some of these were also ameliorated in absence of *MET22*. Our study implies Pus1-dependent Ψ as an RTD-relevant modification that becomes critical for tRNA^Gln^CUG function when different stem regions are destabilized.

## 2. Materials and Methods

### 2.1. Strains and Plasmids

The yeast strains used in this study are listed in Table 1. Yeast was grown in yeast peptone dextrose (YPD) medium or in yeast nitrogen base (YNB) minimal medium lacking specific nutrients such as uracil or individual amino acids [28]. The latter medium was used to select for plasmids, DNA fragments for gene deletion, or to diagnose changes in auxotrophies. Gene deletions were obtained via lithium-acetate-mediated transformation [29] using PCR products in which marker genes from pUG6, pUG27, pUG72, or pUG73 were flanked with 50 nucleotides identical to target genes as described [30]. Correct integration of such gene deletion cassettes was verified using diagnostic PCR according to Reference [30]. Oligonucleotides used are listed in Table S1. *HIS3*-selected plasmids for single copy expression of *SUP70*, *sup70-65*, and *sup70-33* (pSUP70, pSUP70-33, pSUP70-65) were described previously [31]. To study the complementation of *sup70*, we first generated a *SUP70* gene deletion in the presence of pAK01 (*SUP70*-CEN-*URA3*), and then introduced either pSUP70, pSUP70-33, or pSUP70-65 [31]. For multicopy expression of *tQ(UUG)*, the tRNA gene was subcloned from YEplacQ [32] into pRS423 using EcoRI and SalI, creating pRK51. A similar pRS425-based construct has been described [33]. Overexpression of *SUP70* involved pSUP70 2µ [31]. To study the ability to lose pAK01, strains were first streaked from selective histidine- and uracil-free YNB medium to non-selective YPD medium and incubated for 24 h. Next, cells were recovered from the plates, washed with sterile water, and applied to uracil-free YNB and YNB medium containing uracil and 5-fluoro-orotate (5-FOA). Plates were incubated at 30 °C. Rescue of integrated auxotrophic marker genes was done using transient expression of CRE recombinase using constructs and procedures described previously [30].

### 2.2. Growth Phenotype

For phenotyping assays, yeast strains were first grown on solid YPD or plasmid-selective YNB medium for 24 to 36 h. Suspensions of cells were prepared by resuspending cell material recovered from the plates in sterile water. Cell densities were determined photometrically and adjusted to an OD_600nm_ of 1.5. Ten-fold serial dilutions were prepared in sterile water and subsequently spotted on YPD or YNB minimal media. For temperature-sensitivity tests, identical replicates of plates from the same set of cell dilutions were incubated at different temperatures and photographed after 2–4 days.

### 2.3. RNA Isolation and Reverse Transcription

Total RNA was isolated from yeast strains grown to OD_600nm_ = 1 using NucleoZOL (Macherrey-Nagel, Düren, Germany). Two micrograms of total RNA was treated with RNase-free DNase (Thermo scientific, Waltham, USA) in 1× reaction buffer containing MgCl_2_ (reaction volume of 20 µL) for 30 min at 37 °C. After addition of 2 µL 50 mM EDTA, samples were heated to 65 °C for 10 min. For first-strand cDNA synthesis, 0.9 µL of DNase treated samples were incubated with 15 oligo O166 and O1550 (Table S1) in a total volume of 12 µL. Samples were heated to 65 °C for 5 min, chilled on ice, and reaction buffer, RNase inhibitor, dNTPs, and reverse transcriptase added as recommended in the instructions of the RevertAid first strand cDNA synthesis kit (Thermo Scientific). For each RNA sample, a control reaction omitting the reverse transcriptase was prepared. Amplification of cDNAs specific for tRNA^Gly^GCC and *sup70-33* was carried out using 0.5 µL of cDNA or control reaction and oligos O165/O166 or O171/O1550 (Table S1) in a reaction volume of 20 µL with DreamTaq polymerase (Thermo Scientific). Reactions were separated on a 2% agarose gel.

### 2.4. Quantification of Misreading

In vivo mistranslation rates of the CGC (Arg) codon by tRNA^His^GUG were determined using dual luciferase plasmids pDB688 (control) and pDB868 (H245R) [35]. Both plasmids were introduced into BY4741 wild-type, *pus1*, and *pus1 met22* double mutants. Resulting strains were grown in uracil-free YNB overnight at either 30 °C or 37 °C and in the presence or absence of 200 µg/mL paromomycin. Renilla/firefly luminescence was measured using the dual luciferase reporter assay kit (Promega, Fitchburg, USA) and a Glomax luminometer (Promega). Six to eight biological replicates were conducted for each strain and condition and mistranslation rates were calculated by normalizing the firefly H245R activity to the upstream renilla luciferase of the same construct and then normalizing to the activity ratio of the two luciferases from the control construct in the same genetic background.

## 3. Results

### 3.1. Yeast Reporter Strains for CAG Decoding by tRNA^Gln^UUG or tRNA^Gln^CUG

**Figure 1:**
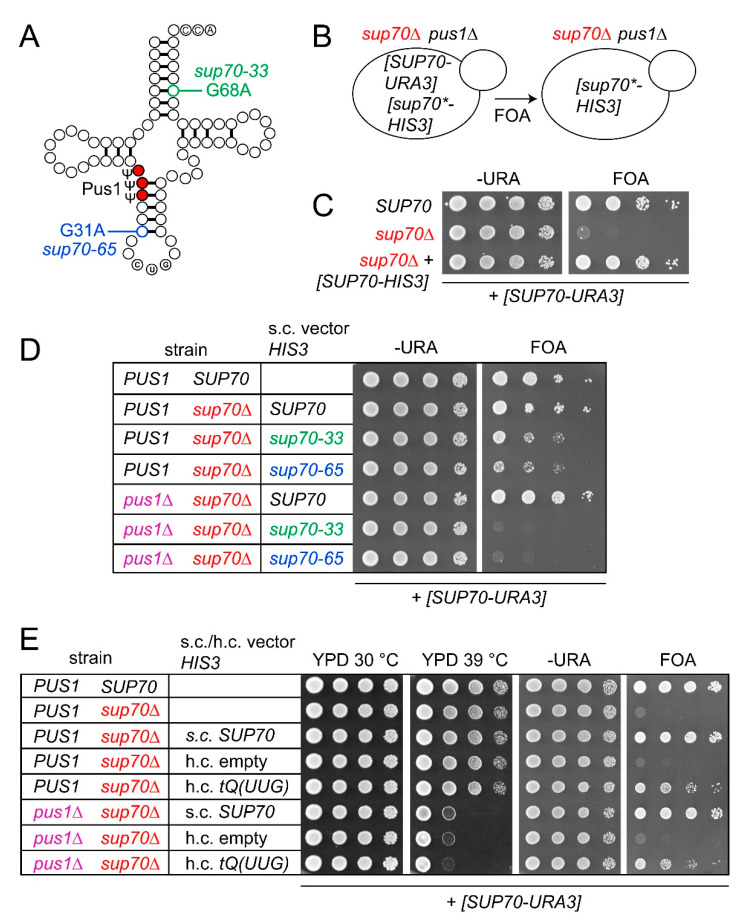
Complementation of *sup70* mutation by *sup70-33*, *sup70-65*, and *tQ(UUG)* in presence and absence of *PUS1*. (**A**) Scheme depicting tRNA^Gln^CUG with base exchanges present in *sup70-33* and *sup70-65* alleles, as well as Pus1-dependent pseudouridylation sites highlighted. (**B**) Outline of plasmid shuffle approach. Yeast strains carrying a deletion of *SUP70* and 5-fluoro-orotate (5-FOA) counter-selectable *SUP70* plasmids [*SUP70-URA3*] were used. In addition, *HIS3*-selected plasmids were introduced, which carry either native *SUP70* or *sup70-33/sup70-65* alleles [sup70*-HIS3]. The complementation ability of the *HIS3*-selected plasmids was determined in the presence or absence of a genomic *PUS1* deletion. (**C**) Proof of principle assay demonstrating inviability of the reporter strain (*sup70* [*SUP70-URA3*]) on 5-FOA medium (FOA) in the absence of native *SUP70* ([*SUP70-HIS3*]). (**D**) Complementation of *sup70* by *SUP70*, *sup70-33*, and *sup70-65* in the presence and absence of *PUS1*. Relevant strain genotype is indicated (strain). s.c.: single copy. (**E**) Complementation of *sup70* by high-copy (h.c.) *tQ(UUG)* or single copy (s.c.) *SUP70* in the presence and absence of *PUS1*. h.c. empty: empty vector.

In yeast, the CAG (Gln) codon decoding tRNA^Gln^CUG is encoded by a single essential gene (*SUP70*) (Figure 1A) [36]. *SUP70* alleles *sup70-33* and *sup70-65* form tRNA^Gln^CUG variants which exhibit reduced stability and charging efficiency in vivo but provide sufficient CAG decoding activity to sustain viability in the absence of native *SUP70* [31,37,38]. These tRNA^Gln^CUG variants contain base exchanges G31A (*sup70-65*) and G68A (*sup70-33*), destabilizing the acceptor- and anticodon-stems, respectively (Figure 1A). Inviability of *sup70* mutants can also be suppressed by overexpression of the inefficient CAG decoder tRNA^Gln^UUG [39]. In order to assess the in vivo CAG decoding potential of tRNA^Gln^UUG, *sup70-33* and *sup70-65* in the absence of Pus1-dependent Ψ, we generated plasmid shuffle strains containing a *sup70* deletion complemented with a counter-selectable *URA3* construct carrying native *SUP70*. To this background, a *PUS1* deletion was introduced as well as *HIS3*-selected constructs providing either *SUP70*, *sup70-33*, or *sup70-65* in single copy (s.c.) or the tRNA^Gln^UUG gene *tQ(UUG)* in multicopy (h.c.). Upon 5-fluoro-orotate (5-FOA)-mediated chase out of the *SUP70-URA3* vector, the *sup70* complementation efficiency of these tRNA genes was inferred by monitoring growth on 5-FOA-supplemented medium (Figure 1B).

As shown in Figure 1C, the inviability of *sup70* mutants with the *SUP70-URA3* plasmid on 5-FOA medium demonstrated that the plasmid indeed could not be lost from a *sup70* strain unless another *SUP70* plasmid (*SUP70-HIS3*), which was retained on 5-FOA medium, was present. While either *SUP70* or the destabilized variants *sup70-33* and *sup70-65* were sufficient for complementation in the wild-type background, mutation of *PUS1* ablated the *sup70* complementation ability of the destabilized variants without affecting complementation by native *SUP70* (Figure 1D). This result indicates that absence of Pus1-dependent Ψ weakened the ability of the destabilized tRNA^Gln^CUG variants to carry out cognate CAG decoding, which is the essential function of *SUP70*. When the complementation of *sup70* by multicopy expression of *tQ(UUG)* was analyzed, no difference between *sup70* and *sup70 pus1* strains was observed (Figure 1E). Since the *pus1* mutation is correlated with temperature sensitivity, we confirmed the presence of this phenotype in the *sup70 pus1* strains (Figure 1E). Hence, in contrast to the tRNA^Gln^CUG variants providing reduced CAG-decoding potential, the inefficient CAG-decoding function of tRNA^Gln^UUG was not further impaired in the absence of Pus1.

### 3.2. Role of *MET22* in *pus1* and *sup70-33/sup70-65* Phenotypes

**Figure 2:**
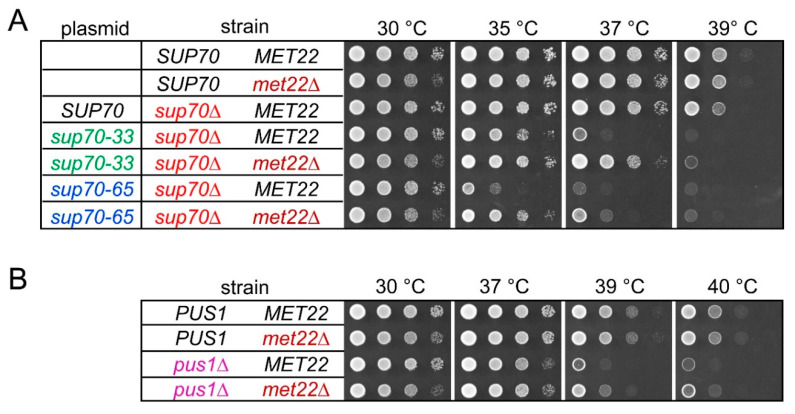
Thermosensitive growth phenotypes of *sup70-65*, *sup70-33*, and *pus1* strains are partly suppressed by deletion of *MET22*. (**A**) Growth of wild-type (*SUP70 MET22*), *met22*, and *sup70-33/sup70-65* mutants with and without *met22* deletion at elevated temperatures. Mutants carrying *sup70-33* or *sup70-65* as sole source of tRNA^Gln^CUG were generated by complementing a genomic *sup70* deletion (*sup70*∆) with plasmid-borne *sup70-33* or *sup70-65*, respectively. For control purposes, native *SUP70* was provided from the same vector backbone (*sup70∆ SUP70*). Plasmids used are indicated in the column labeled “plasmid”. (**B**) Growth of wild-type (*PUS1 MET22*), *met22* and *pus1* single mutants, and *pus1 met22* double mutants at elevated temperatures.

Previously, we observed a strong temperature-sensitive growth (*ts*) phenotype in yeast cells relying on the *sup70-65* variant for CAG decoding [40]. We reconfirmed this result and further demonstrated that *sup70-33* is also linked to a robust *ts* phenotype (Figure 2A). Similarly, mutation of *PUS1* is correlated with a milder *ts* phenotype that slows growth at 38 °C and above [15] (Figure 2B). In some cases, the absence of core modifications in tRNA may trigger degradation by rapid tRNA decay (RTD), which is induced by mild heat stress [18]. To test whether the observed *ts* phenotypes in *sup70-33*, *sup70-65*, and *pus1* mutants could be attributable to RTD, we combined these mutations with a *met22* deletion and compared growth at elevated temperatures between the *MET22* parental strains and the *met22* derivatives. As shown in Figure 2A, the *ts* phenotypes of strains carrying *sup70-33* or *sup70-65* as the sole *SUP70* variant were partially rescued by deletion of *MET22*. While the *sup70-65* strain was severely growth-impaired at 35 °C, the *sup70-65 met22* strain exhibited much more robust growth in this condition. A similar improvement was seen for the *sup70-33 met22* strain at 37 °C (Figure 2A). Likewise, an improvement of growth at temperatures above 37 °C was observable in the *pus1 met22* double mutant as compared to the single mutants (Figure 2B). In contrast to *pus1*, we found that loss of the Deg1 Ψ synthase modifying U38 and U39 [6] was linked to a *MET22*-independent *ts* phenotype (Figure S1). Thus, destabilized tRNA^Gln^CUG variants and *pus1* mutants exhibit *ts* growth phenotypes that could in part result from activation of RTD, while the phenotype associated with *deg1* mutation may result from RTD independent effects. We further tested whether the *ts* phenotype of *pus1* mutants might be mainly due to functional defects in tRNA^Gln^ by overexpressing both isoacceptor tRNAs. However, the presence of elevated copy numbers of *tQ(CUG)* and *tQ(UUG)* alone or in combination was insufficient to suppress the *pus1* phenotype (Figure S2).

### 3.3. Effect of *met22* Mutation on Abundance of *sup70-33* tRNA

**Figure 3:**
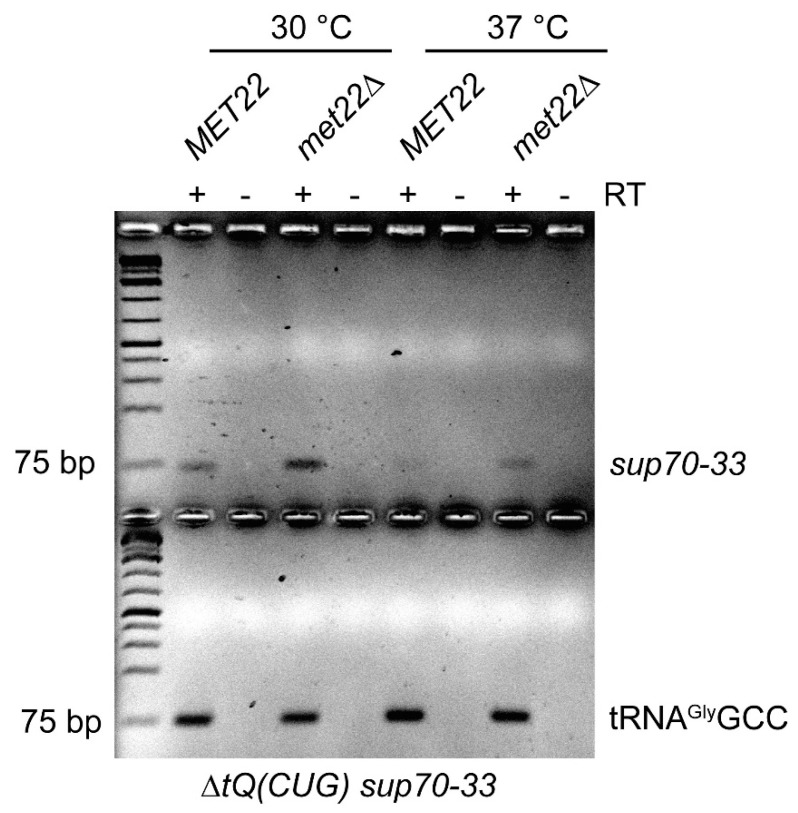
Impact of *met22* mutation on *sup70-33* abundance. Total RNA was isolated from *sup70-33* mutants with or without the *met22* mutation, grown at 30 °C or shifted to 37 °C for 5 h as indicated. Identical amounts of RNA were reverse-transcribed (RT+) to cDNA using oligonucleotides specific for *sup70-33* and tRNA^Gly^GCC, respectively. For control purposes, identical reactions were carried out omitting the reverse transcriptase (RT-). Low-cycle-number PCR was used to amplify cDNAs from *sup70-33* and tRNA^Gly^GCC and reactions were separated on a 2% agarose gel. The 75 bp band of the DNA marker used is indicated.

The suppression of *ts* phenotypes of the destabilized *SUP70* variants by *met22* suggested the involvement of RTD-like mechanisms in the destabilization process. Since not only loss of modifications but also destabilized stem regions in tRNA can trigger RTD [41,42,43,44], we further explored the possibility that *sup70-33* tRNA may be destabilized by this surveillance mechanism. We utilized a reverse-transcriptase-based assay that specifically amplified cDNA from the *sup70-33* tRNA without detecting the nearly identical tRNA^Gln^UUG (Figure S3). This assay made use of the *sup70-33*-specific G68A base change which is not present in tRNA^Gln^UUG. Next, we employed this assay to monitor cDNA generation efficiency with identical amounts of total RNA derived from *sup70-33* strains with and without the *met22* mutation either grown at 30 °C or shifted to 37 °C for 5 h. As shown in Figure 3, the *sup70-33* cDNA signal declined in the 37 °C culture as compared to the 30 °C culture, while a control cDNA for tRNA^Gly^GCC remained unaffected. Further, we observed elevated cDNA formation for *sup70-33* when *MET22* was mutated at both 30 °C and 37 °C. These results revealed that *sup70-33* is destabilized in a temperature- and *MET22*-dependent manner, conditions commonly observed for well-studied RTD cases [26,41,42].

### 3.4. Suppression of Synthetic Lethal Genetic Interaction between Destabilized *sup70* Variants and *PUS1*

**Figure 4:**
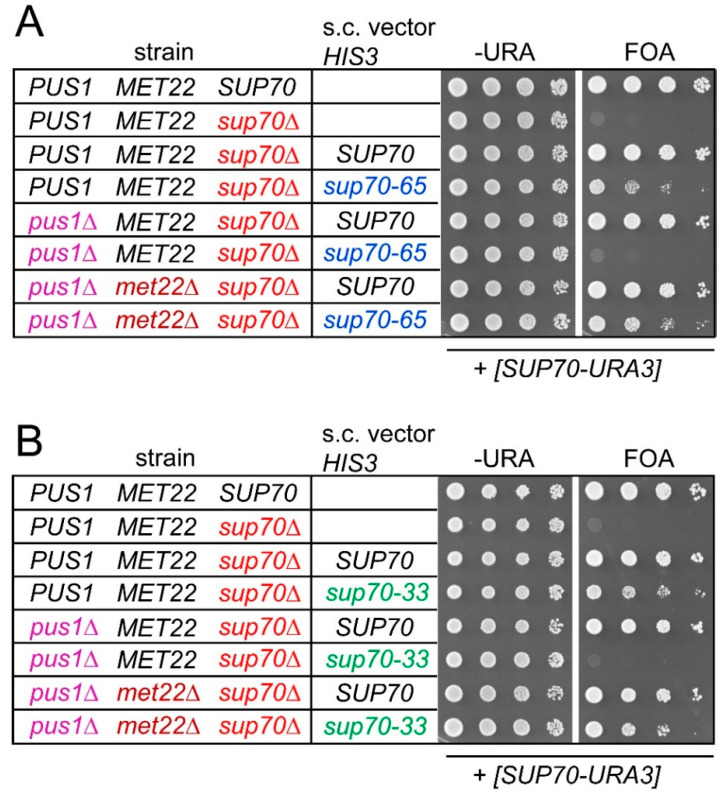
Synthetic lethal interaction between *pus1* and *sup70-33/sup70-65* was suppressed by *met22* mutation. (**A**) Plasmid shuffle approach (as in Figure 1) for analysis of *sup70* complementation by *SUP70* or *sup70-65* in absence of *PUS1* (*pus1*∆), *MET22* (*met22*∆), or both. All strains carried the [*SUP70-URA3*] plasmid. Indicated strains additionally carried *HIS3*-plasmids (s.c. vector *HIS3*) providing *SUP70* or *sup70-65* as indicated. Top: wild-type BY4741. (**B**) Plasmid shuffle approach for analysis of *sup70* complementation by *SUP70* or *sup70-33* in absence of *PUS1* (*pus1*∆), *MET22* (*met22*∆), or both.

Since negative consequences of *PUS1* deletion and either *sup70-33* or *sup70-65* are individually suppressed by deletion of the RTD factor *MET22* (Figure 2), we investigated whether the synthetic lethality caused by combining the tRNA modification defect with the *sup70* variants was suppressible by *met22* mutation as well. We used the plasmid shuffle approach described above (Figure 1) in strains that carried either a *MET22* wild-type allele or a genomic deletion. As shown in Figure 1D, *pus1 sup70* double mutants with the *SUP70-URA3* construct were able to grow on 5-FOA medium when wild-type *SUP70* was provided by a second plasmid, but not with *sup70-33* or *sup70-65*. However, we observed that this requirement for native *SUP70* was bypassed upon deletion of *MET22* (Figure 4). In the triple *pus1 sup70 met22* mutant containing the *SUP70-URA3* plasmid, either form of *SUP70* (including the destabilized variants) permitted growth on 5-FOA medium (Figure 4). Thus, the synthetic lethality of *pus1 sup70-33/sup70-65* was suppressed by the RTD-ablating *met22* mutation.

### 3.5. Role of *PUS1* and *MET22* in UAG Misreading by *sup70-65* at Different Temperatures

**Figure 5:**
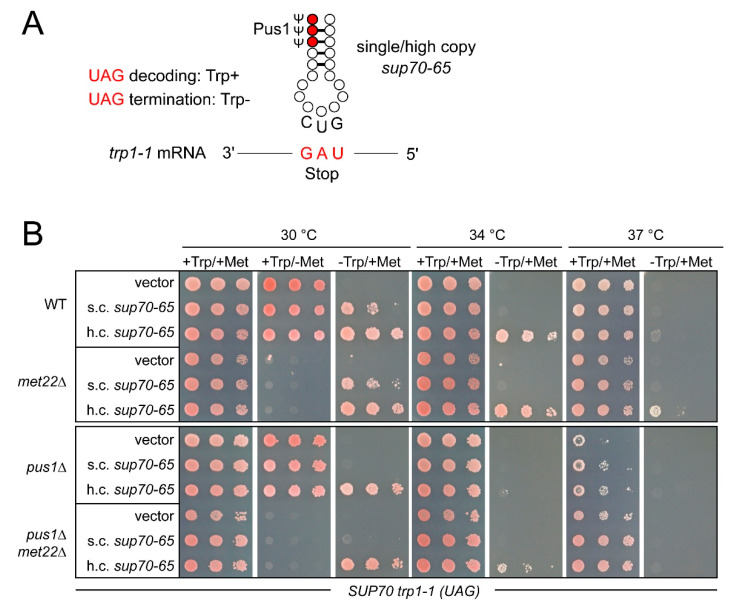
UAG decoding by *sup70-65* in absence of *PUS1* and *MET22*. (**A**) Scheme depicting the first codon position U/G error in the decoding of UAG stop codons by *sup70-65*. In the *trp1-1* allele, a premature UAG codon abrogates translation of the *trp1-1* mRNA, leading to tryptophan auxotrophy (Trp-). In the presence of *sup70-65* in single or high copy numbers, UAG readthrough occurs, leading to a partial or full reversion of tryptophan auxotrophy (Trp+) associated with the *trp1-1* allele. (**B**) Assessment of UAG readthrough by *sup70-65* in single copy (s.c.) or high copy (h.c.) numbers at different temperatures and in the absence of *MET22*, *PUS1*, or both. +Trp/+Met refers to minimal medium supplemented with both methionine and tryptophan (control), -Trp/+Met medium allowed UAG readthrough to be determined, and +Trp/-Met medium was employed as a phenotypic control for the presence of the *met22* mutation that causes methionine auxotrophy.

The *sup70-65* variant of tRNA^Gln^CUG exhibits a well characterized ability to misread UAG stop codons via first codon position U/G wobble pairing [31,38]. The efficiency of this decoding event can be scored in yeast strains carrying a premature UAG stop codon within the tryptophan biosynthesis gene *TRP1* (*trp1-1* allele). In such strains, UAG misreading reverted the inability to grow on tryptophan-free medium (trp-) (Figure 5A). In order to assess how elevated temperature and loss of Pus1 influenced the efficiency of *sup70-65* in UAG misreading, we introduced *pus1* and *met22* deletions into a reporter strain carrying the *trp1-1* allele and subsequently expressed *sup70-65* in single copy (s.c.) and high copy (h.c.) numbers. In these strains, the native *SUP70* allele was present, allowing the effect of *pus1* mutation on *sup70-65* function to be studied. As described earlier, high-copy expression of *sup70-65* is required to enable robust growth on trp-, whereas single-copy expression permits substantially weaker growth (Figure 5B) [40]. When we assessed the impact of elevated temperature, we observed a gradual decline in UAG suppression enabling growth on trp- medium. At 34 °C, no growth was observable with s.c. expression of *sup70-65* and at 37 °C, even h.c. *sup70-65* failed to provide tryptophan prototrophy (Figure 5B). Since the reporter strains grew normally on tryptophan-supplemented control plates, these differences were attributable to changes in UAG decoding required for Trp1 expression from the *trp1-1* allele. Thus, mildly elevated temperature reduced the UAG decoding potential of *sup70-65*.

When *sup70-65-*mediated UAG decoding was scored in the presence of the *pus1* mutation, a clear negative impact of the modification defect became apparent (Figure 5B). In contrast to the wild-type at 30 °C, s.c. *sup70-65* was insufficient in *pus1* mutants to enable growth on trp- medium. At elevated temperatures (34 °C and 37 °C), neither the s.c. nor the h.c. expression of *sup70-65* resulted in an ability of the *pus1* mutant to grow on trp- medium. Hence, *sup70-65-*mediated UAG decoding was impaired by the loss of *PUS1*. The differences in growth on trp- at 30 °C and 34 °C were indeed due tryptophan auxotrophy and not due to general growth defects, since normal growth was observed on the tryptophan-supplemented control plates (Figure 5B). Only at 37 °C did a mild growth inhibition of the *pus1* mutant become apparent.

Interestingly, the *MET22* deletion resulted in a partial suppression of the UAG-decoding defects observed. At 34 °C, *pus1 met22* double mutants carrying h.c. *sup70-65*, but not the *pus1* single mutant with the same vector, were able to grow weakly on trp- medium. Weak growth on this medium at 37 °C was also observed for the *met22* single mutant, but not for the wild-type carrying h.c. *sup70-65*. These observations suggest that modest temperature increases gradually impaired the UAG-decoding ability of *sup70-65*. This effect was aggravated in the absence of Pus1 and partially suppressed by the RTD-ablating *met22* mutation. Hence, not only the cognate CAG-decoding ability of *sup70-65*, but also the efficiency in UAG mistranslation appeared to be affected by Pus1 in a similar way.

### 3.6. Role of *PUS1* and *MET22* in Paromomycin-Induced Mistranslation

Since Pus1-dependent pseudouridine is present in several tRNAs, it may positively affect not only *sup70* variants but also other tRNAs, and thereby influence translational fidelity. To assess this potential role of Pus1, we utilized a well characterized dual luciferase reporter system to quantify basal and paromomycin-induced mistranslation [35,45]. In this assay, incorporation of histidine by tRNA^His^GUG is measured at the CGC (Arg) codon in firefly luciferase. It was previously shown that this misreading event is triggered in vivo by the aminoglycoside paromomycin [35,45]. Since the natural CGC decoder tRNA^Arg^ICG contains Pus1-dependent Ψ, but not the mistranslating tRNA^His^GUG [14], this assay provided an opportunity to determine the effect of the modification on the competition between both tRNAs for CGC decoding.

**Figure 6:**
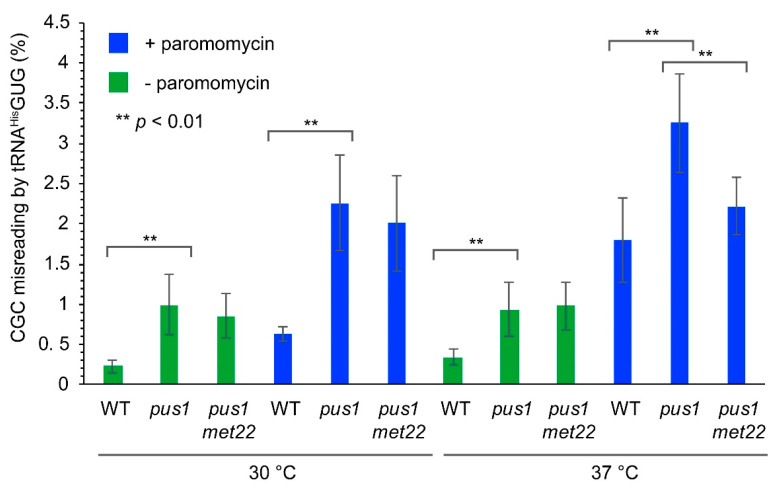
CGC misreading levels in *pus1* mutants. Indicated strains were each transformed with two dual luciferase constructs (renilla/firefly control pDB688 and renilla/firefly-H245R pDB868). Strains were cultivated at the indicated temperature and in the presence or absence of 200 µg/mL paromomycin. At least six independent measurements were conducted for each strain/condition Two-tailed Students’ *t*-test was utilized to determine statistical significance (**: *p* < 0.01).

We introduced a dual luciferase (renilla/firefly) construct carrying a CAC (His245) to CGC (Arg245) codon substitution in the firefly luciferase gene, as well a control construct expressing unmodified luciferases into the wild-type and *pus1* mutants. The dual luciferase reporter and normalization against the control dual luciferase construct without the H245R substitution allowed the measurement of specific CGC misreading rates by tRNA^His^GUG and eliminated any potential impact of mRNA abundance changes on the calculated error rates [35]. Interestingly, the absence of *PUS1* caused a significant increase (4.3-fold) in basal CGC misreading, which was, however, not suppressed by additional mutation of *MET22* (Figure 6). When paromomycin was added during cultivation (200 µg/mL), we observed increased in error rates in both wild-type and the *pus1* mutant. The latter again showed significantly increased (~3.6-fold) CGC mistranslation as compared to the wild-type in the same condition, without a decline in the *pus1 met22* double mutant. However, when the aminoglycoside was applied at an elevated temperature (37 °C), CGC misreading in the *pus1* mutant was further increased and this effect was dependent on the presence of *MET22*, since misreading rates in the *pus1 met22* double mutant were significantly lower (Figure 6). These results revealed that Pus1-dependent Ψ is involved in the prevention of translational errors at the CGC codon, likely due to a positive contribution of the modification to the efficiency of the natural CGC decoder tRNA^Arg^ICG. Since *met22* effects were observed for paromomycin-induced mistranslation at elevated temperature, the positive role of Pus1 may in part involve protection from RTD-mediated decay.

## 4. Discussion

Pseudouridine represents the most abundant tRNA modification and, in general, it is thought to stabilize the tertiary structure of the tRNA and improve base stacking [1]. However, the occurrence of this modification in multiple positions, including loop and stem regions of the tRNA, may have distinct functional consequences. We focused on the role of Pus1-dependent pseudouridylation for tRNA function in vivo. While in yeast, Pus1 and its modification are dispensable per se [11], it becomes critical when tRNA^Gln^CUG is compromised by mismatches in the acceptor (*sup70-33*) or anticodon stems (*sup70-65*). These *sup70* alleles encode destabilized forms of tRNA^Gln^CUG which provide reduced ability to carry out CAG decoding as the essential function of *SUP70* [31,37,38]. When combining a *pus1* mutation with either *sup70-33* or *sup70-65*, synthetic lethality occurs, which can be attributed to a further aggravated CAG-decoding defect. A similar synthetic lethal genetic interaction was previously demonstrated for *pus1* and the *slc58* allele, encoding another tRNA^Gln^CUG variant with a mismatch destabilizing the T-stem [16]. Hence, Pus1-dependent pseudouridylation likely represents a stabilizing factor that becomes particularly relevant for tRNA^Gln^CUG function when stability of the tRNA is compromised otherwise. Similarly, Deg1-dependent psedouridylation at position 38 (anticodon loop) becomes critical for tRNA^Gln^CUG function only when the anticodon stem destabilizing *sup70-65* mutation is present [40]. In contrast to the Deg1-dependent Ψ38 [46], Pus1-dependent Ψ26-28 does not appear to be crucial for the major Gln isoacceptor tRNA^Gln^UUG, as *tQ(UUG)* overexpression suppressed the inviability of a *sup70* mutant in absence of *PUS1*. Since *tQ(UUG)* is very inefficient in CAG decoding [39], any further impairment should be detrimental, which is, however, not observed in absence of Pus1. Hence, the different Ψ formed by Pus1 and Deg1 are of different importance for the two Gln isoacceptor tRNAs. It was speculated previously that Deg1-dependent Ψ is specifically important due to its potential contribution to the stabilization of the anticodon loop [40,47]. Since Pus1-dependent Ψ is localized in the anticodon stem of tRNA^Gln^, a distinct functional contribution appears plausible. The tRNA overexpression approach shown in Figure S2 suggests that additional tRNAs or other RNA species modified by Pus1 may contribute to the mutant phenotype.

Interestingly, in other tRNAs, stem mismatches and loss of different modifications outside of the anticodon loop may trigger rapid tRNA decay (RTD) [18]. Temperature is recognized as an important factor in RTD, since in several hypomodified or structurally destabilized tRNAs, full decay is induced after modest temperature increases [18,41,42,48]. However, neither of the described tRNA^Gln^CUG-destabilizing mutations nor the Pus1-dependent Ψ were known to represent RTD-relevant factors. Since tRNA^Ser^ and tRNA^Tyr^ derivatives with stem mismatches become susceptible to RTD [41,42,43,44], a similar effect in *sup70-33* and *sup70-65* appeared to be possible. Thus, we investigated the potential suppression by *met22* mutation, which broadly inhibits RTD [26,41,43,44,48].

Loss of Pus1 or destabilization of tRNA^Gln^CUG is correlated with increased temperature sensitivity and we indeed observed partial suppression of these phenotypes by *met22* mutation. In addition, the synthetic lethality caused by combining the *pus1* defect and acceptor/anticodon stem mismatches in tRNA^Gln^CUG was suppressed by *met22* mutation, which suggests that the observed phenotypes are linked to RTD. In support of this idea, we demonstrated that the *sup70-33* variant is further destabilized in vivo by mild heat and this effect is suppressible by *met22* mutation. Since the *pus1*- but not the *deg1-*linked *ts* phenotype was suppressed by *met22* mutation, Deg1-dependent Ψ located in the anticodon loop and anticodon stem base may be much less RTD-relevant as compared to the Pus1-dependent Ψ found in multiple positions. Further work will be required to identify additional tRNAs that rely on Pus1-dependent Ψ for protection against RTD.

In addition to its essential role in CAG decoding, *sup70-65* can also misread UAG stop codons via first codon position U/G wobble, possibly due to a change in the structure and orientation of the anticodon loop in response to the mutation in the anticodon stem [31]. Our diagnosis of UAG suppression by *sup70-65* revealed a clear negative influence of mildly elevated temperature. Loss of Pus1 had a similar negative effect on UAG decoding by *sup70-65*, indicating that Pus1 positively contributes to the UAG mistranslating ability of the tRNA. Thus, it appears that Pus1 positively contributes to both misreading and cognate decoding. Similar effects were described for wobble uridine modifications, which support both the decoding efficiency in normal translation and near-cognate misreading [49,50,51,52,53], pointing to a general positive role of different tRNA modifications in tRNA function. Deg1-dependent Ψ was also found to improve stop codon readthrough by natural suppressor tRNAs [54], which may be consistent with an important role of the modification in anticodon loop stabilization supporting both regular decoding and stop codon misreading. In contrast, no effect of Pus1 on UAG readthrough by natural suppressor tRNA was observed [54], which is consistent with our data showing that Pus1 becomes critical for tRNA^Gln^CUG function specifically when stem regions are destabilized.

When the effect of *met22* mutation on UAG misreading by *sup70-65* was studied, mild suppression of the negative effects of elevated temperature and *pus1* mutation were observed. However, suppression in *met22* and *pus1 met22* strains was incomplete, suggesting that even when RTD is inhibited, both increased temperature and loss of Pus1 have negative consequences for *sup70-65* function in UAG mistranslation. We assume that Pus1 may fulfil additional and potentially more direct decoding relevant functions in tRNA beyond the protection from RTD. Interestingly, diploid yeast strains carrying homozygous *sup70-65* or *sup70-33* alleles display disturbed nitrogen signaling and inappropriate filamentous growth responses [31,38,55]. If these effects are linked to the destabilization of tRNA^Gln^CUG, they may be suppressed by inhibition of RTD, similarly to the *ts* phenotypes investigated in this study. Nitrogen signaling defects might also contribute to some extent to the *ts* phenotypes of the *sup70* alleles, in addition to the established impairment in CAG decoding.

The presence of Pus1-dependent Ψ in additional tRNAs and the absence of the suppression of the *pus1 ts* phenotype by tRNA^Gln^ overexpression argues for a positive role of the modification in additional tRNAs. Indeed, the absence of Pus1 significantly elevated misreading of CGC (Arg) codons by tRNA^His^. In this misreading event, the normal CGC decoder tRNA^Arg^ICG contained Pus1-dependent Ψ, but not the mistranslating tRNA^His^ [14]. Hence, in tRNA^Arg^ICG, Pus1-dependent pseudouridylation likely represents an important factor to support competition against a tRNA carrying an incorrect near-cognate anticodon (U/G in 2nd codon position). Since the mistranslation rates were similar at both 30 °C and 37 °C and in both conditions were unaffected by *met22* mutation, the positive effect of Pus1 in tRNA^Arg^ICG may involve other mechanisms than protection from RTD-mediated decay. However, at 37 °C, a further increase of paromomycin-induced mistranslation in the *pus1* mutant relative to the wild-type under the same conditions was observed. In contrast to basal mistranslation, this effect was suppressed in the *pus1 met22* double mutant. This result suggests that at elevated temperature, Pus1-dependent Ψ limits aminoglycoside-induced mistranslation in part via RTD-protective effects of the modification. We assume that Pus1 contributes to the efficiency of the tRNA^Arg^ICG in different ways, presumably including RTD-dependent and -independent mechanisms. Further work will be required to define the effect of the abundant Pus1-dependent tRNA modification on the in vivo function of additional tRNAs and to distinguish between RTD-dependent and -independent effects.

## 5. Conclusions

The importance of Pus1-dependent pseudouridylation for specific in vivo decoding events was analyzed. We focused on tRNA^Gln^CUG, since this tRNA is essential in yeast and encoded by a single-copy gene, facilitating genetic approaches. Mutations destabilizing the anticodon or acceptor stem of tRNA^Gln^CUG resulted in synthetic lethality upon combination with a *PUS1* deletion, suggesting that the essential function in CAG decoding by these tRNA variants cannot be maintained in absence of Pus1-dependent Ψ. Phenotypes associated with *pus1* mutation, destabilized tRNA^Gln^CUG, and the negative genetic interaction of both were suppressed by mutation of the rapid tRNA decay gene *MET22*. Pus1-dependent Ψ was also required for efficient UAG mistranslation by a tRNA^Gln^CUG variant, revealing a general positive role of the modification in tRNA function. Consistent with this, we demonstrated that Pus1-dependent Ψ improves the ability of a distinct tRNA to compete against mistranslation by a non-synonymous near-cognate tRNA. Our study provides insight into the contribution of Pus1-dependent pseudouridylation to in vivo tRNA function, which likely includes, but is not limited to the prevention of rapid tRNA decay.

## Figures and Tables

**Table 1:** Strains used in this study.

Strain	Genotype	Reference
BY4741	MATa, *his3, leu2, met15, ura3*	Euroscarf, Frankfurt
met22	BY4741 *met22::KANMX4*	Euroscarf, Frankfurt
pus1	BY4741 *pus1::KANMX4*	Euroscarf, Frankfurt
W303-1B	MATα *leu2-3,112 trp1-1 can1-100 ura3-1 ade2-1 his3-11,15*	[34]
RK134	BY4741 *tQ(CUG)::KlLEU2* pSUP70-65	this work
RK135	BY4741 *tQ(CUG)::KlLEU2* pSUP70-33	this work
RK143	BY4741 *tQ(CUG)::KlLEU2* pSUP70	this work
RK172	BY4741 *tQ(CUG)::KlLEU2* pAK01	this work
RK289	BY4741 *tQ(CUG)::loxP* pAK01	this work
RK356	BY4741 *tQ(CUG)::KlLEU2* pRK51	this work
RK643	BY4741 *tQ(CUG)::KlLEU2* pSUP70-33 *met22::KlURA3*	this work
RK644	BY4741 *tQ(CUG)::KlLEU2* pSUP70-65 *met22::KlURA3*	this work
RK645	BY4741 *tQ(CUG)::loxP* pAK01 *pus1::KlLEU2*	this work
RK648	BY4741 *tQ(CUG)::loxP* pSUP70	this work
RK649	BY4741 *tQ(CUG)::loxP* pSUP70-33	this work
RK650	BY4741 *tQ(CUG)::loxP* pSUP70-65	this work
RK651	BY4741 *tQ(CUG)::loxP* pSUP70 *pus1::KlLEU2*	this work
RK655	BY4741 *tQ(CUG)::loxP* pAK01 *pus1::KlLEU2 met22::KANMX4*	this work
RK666	BY4741 *tQ(CUG)::loxP* pAK01 *met22::KlLEU2*	this work
RK668	BY4741 *tQ(CUG)::loxP* pRK51	this work
RK669	BY4741 *tQ(CUG)::loxP* pRK51 *pus1::KlLEU2*	this work
RK452	W303-1B *pus1::KlLEU2*	this work
RK703	W303-1B *met22::KlURA3*	this work
RK704	W303-1B *pus1::KlLEU2 met22::KlURA3*	this work
RK499	BY4741 *pus1::SpHIS5*	this work
RK567	BY4741 *pus1::SpHIS5 met22::KlLEU2*	this work
RK475	BY4741 *deg1::SpHIS5*	this work
RK658	BY4741 *deg1::SpHIS met22::KlLEU2*	this work

## Supplementary Materials

The following are available online at https://www.mdpi.com/2218-273X/10/5/729/s1, Figure S1: Thermosensitive growth of *deg1* mutants is not rescued by *met22* mutation., Figure S2: tRNA overexpression in *pus1* mutants, Figure S3: Specificity of *sup70-33* detection. Table S1: Oligonucleotides used in this study.
