# Supplementary material for: Impact of Pus1 Pseudouridine Synthase on Specific Decoding Events in Saccharomyces cerevisiae
Source: Biomolecules. 2020 May 7;10(5):729. doi: 10.3390/biom10050729 (PMC7277083; doi:10.3390/biom10050729)
Supplement: Supplementary file 1 [file biomolecules-10-00729-s001.pdf]

Supplementary information

Table S1. Oligonucleotides used in this study.

Name	Sequence (5′-3′)	Purpose
O30	AGGTTCCATAAAACCGGAAGTTTTAGTGTACAC TAACAACAGAAGAAAAACAGCTGAAGCTTCGT ACGC	Deletion of *SUP70*
O31	AAAAAAAAAAATGATGGTTTAAATTTCGTAAA ATACGAAAAATGAAGGGAGCATGCATAGGCC ACTAGTGGATCTG	Deletion of *SUP70*
O28	ATGAGGAGAGCTTCTACTA	Verification of *SUP70* deletion
O29	GACAAGTGCTCATCATTATG	Verification of *SUP70* deletion
O664	ATAAAGGACAATAAAGTGCTAGTAAATAACAA TTATAAGTGATATCAAGGCAGCTGAAGCTTCGT ACGC	Deletion of *PUS1*
O665	ATGTCAATGCCTTAGAAATTAAAGTTGGTAAG AAAGAAGGAAAGGGCAACGCATAGGCCACTA GTGGATCTG	Deletion of *PUS1*
O666	GATGCGGGTAACTATTAGCC	Verification of *PUS1* deletion
O667	GCGCAATGAGCTTTCCAAGG	Verification of *PUS1* deletion
O240	TTAGTAAGTAAGAAGTTTAAAGACAACTCAGA AGACATCAGCACTTTACTCAGCTGAAGCTTCGT ACGC	Deletion of *MET22*
O241	TATATGTACTCATATATTTATGTCTATCAATAAA GTAAAATATATGTTATGCATAGGCCACTAGTGG ATCTG	Deletion of *MET22*
O242	TGCGCGAATGACTCAGACG	Verification of MET22 deletion
O243	GAGATGCCGTCATCGTAGGG	Verification of MET22 deletion
O165	GCGCAAGTGGTTTAGTGGT	tRNAGlyGCC RT-PCR
O166	TGCGCAAGCCCGGAATCGAAC	tRNAGlyGCC RT-PCR
O171	GGTCCTATAGTGTAGTGG	sup70-33 RT-PCR
O1550	GGTCTCACCCGGATTCGAACT	sup70-33 RT-PCR

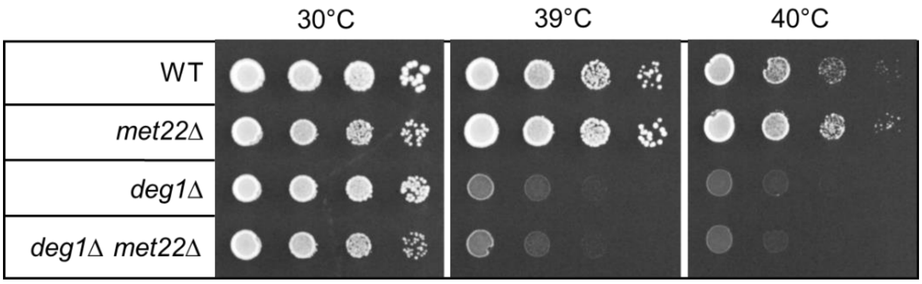

Figure S1. Thermosensitive growth of *deg1* mutants is not rescued by *met22* mutation. Growth of wild type (WT), *met22* (*met22Δ*) and *deg1* single mutants (*deg1Δ*) and *deg1 met22* double mutants (*deg1Δ met22Δ*) at 30°C, 39°C and 40°C was analyzed by serial dilution spot assay.

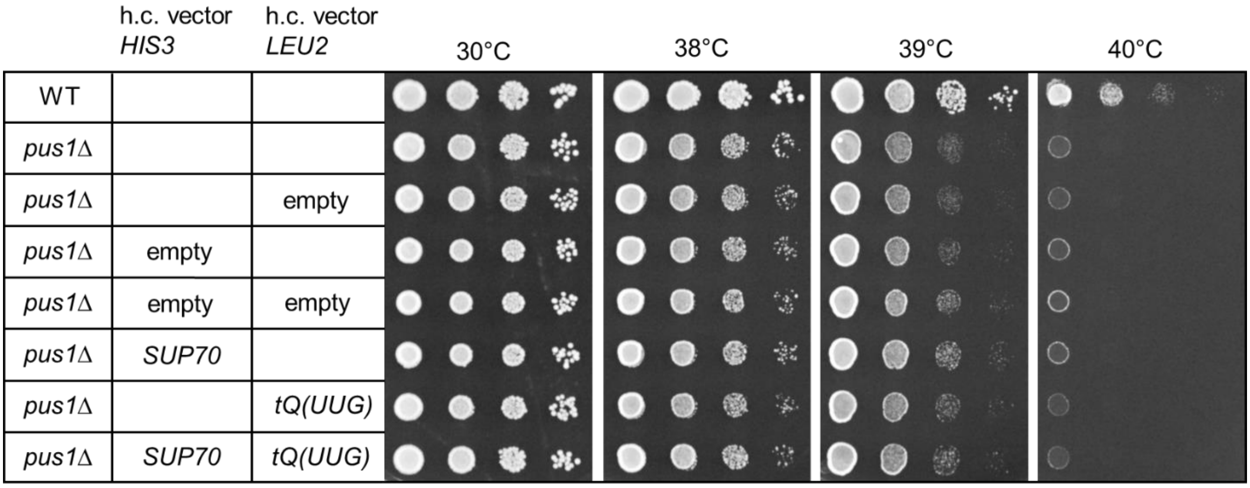

Figure S2. tRNA overexpression in *pus1* mutants. Growth of wild type (WT) and *pus1* mutants (*pus1*Δ) carrying high copy vectors (h.c. *HIS3* and h.c. *LEU2*) with *SUP70* or *tQ(UUG)* genes (pSUP70 2μ and pRK55) was assayed by serial dilution spot assay. empty: empty vector controls.

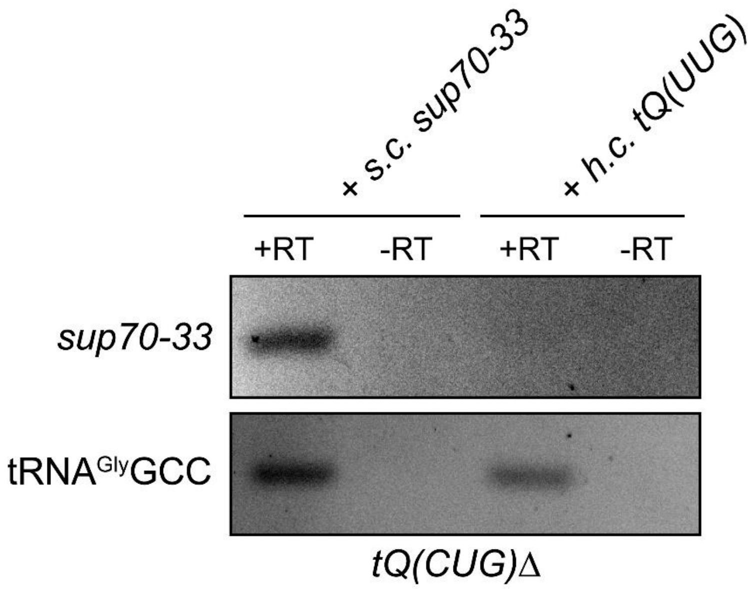

Figure S3. Specificity of *sup70-33* detection. Total RNA was isolated from strains carrying the genomic *sup70* deletion (*tQ(CUG)Δ*) and either single copy *sup70-33* (s.c. *sup70-33*) or multi copy *tQ(UUG)* (h.c. *tQ(UUG)*). Identical amounts of RNA were revere-transcribed (RT+) to cDNA using oligonucleotides specific for *sup70-33* and tRNA^{Gly}GCC, respectively. For control purposes, identical reactions were carried out omitting the reverse transcriptase (RT-). Low cycle number PCR was used to amplify cDNAs from *sup70-33* and tRNA^{Gly}GCC. The absence of a *sup70-33* cDNA product in the *sup70* h.c. *tQ(UUG)* strain demonstrates specificity of *sup70-33* detection.
